# *LC_Glucose-Inhibited Division Protein* Is Required for Motility, Biofilm Formation, and Stress Response in *Lysobacter capsici* X2-3

**DOI:** 10.3389/fmicb.2022.840792

**Published:** 2022-03-17

**Authors:** Dan Zhao, Hong Wang, Zhiyuan Li, Shengnan Han, Chao Han, Aixin Liu

**Affiliations:** Shandong Provincial Key Laboratory of Agricultural Microbiology, College of Plant Protection, Shandong Agricultural University, Tai’an, China

**Keywords:** *Lysobacter capsici*, glucose-inhibited division protein, motility, biofilm formation, colonization, stress response

## Abstract

Glucose-inhibited division protein (GidA) plays a critical role in the growth, stress response, and virulence of bacteria. However, how *gidA* may affect plant growth-promoting bacteria (PGPB) is still not clear. Our study aimed to describe the regulatory function of the *gidA* gene in *Lysobacter capsici*, which produces a variety of lytic enzymes and novel antibiotics. Here, we generated an *LC_GidA* mutant, MT16, and an *LC_GidA* complemented strain, Com-16, by plasmid integration. The deletion of *LC_GidA* resulted in an attenuation of the bacterial growth rate, motility, and biofilm formation of *L. capsici*. Root colonization assays demonstrated that the *LC_GidA* mutant showed reduced colonization of wheat roots. In addition, disruption of *LC_GidA* showed a clear diminution of survival in the presence of high temperature, high salt, and different pH conditions. The downregulated expression of genes related to DNA replication, cell division, motility, and biofilm formation was further validated by real-time quantitative PCR (RT–qPCR). Together, understanding the regulatory function of GidA is helpful for improving the biocontrol of crop diseases and has strong potential for biological applications.

## Introduction

*Lysobacter* spp. are bacteria natively present in the rhizosphere, water, and some extreme conditions (Park et al., 2008; Fang et al., 2020). In recent years, species, such as *Lysobacter enzymogenes*, *Lysobacter antibioticus*, and *Lysobacter capsici*, have attracted much interest for their antimicrobial activities, and they are regarded as effective biocontrol agents of plant diseases (Yu et al., 2018; Afoshin et al., 2020). For example, heat stable antifungal factor (HSAF), isolated from *L. enzymogenes* C3, has been exhibited to be inhibitory activities against a wide range of fungal species (Yu et al., 2007). Compared to *L. enzymogenes*, much less is known about the biological features of *L. capsici*. The *L. capsici* AZ78 genome has a gene pool that allows it to successfully interact with plant pathogenic microorganisms and environmental factors, providing a genetic framework for detailed analysis of potential biocontrol mechanisms of plant pathogens (Puopolo et al., 2016). In addition, the effective antifungal effect of *L. capsici* AZ78 and *L. capsici* PG4 has been shown (Puopolo et al., 2010; Brescia et al., 2020). Twenty-two volatile organic compounds to be produced by *L. capsici* AZ78, that contribute to biological control of soilborne plant pathogens (Vlassi et al., 2020). Overall, the species of *L. capsici* has considerable potential for biocontrol of plant pathogenic microorganisms.

tRNA modification ensures efficient and accurate protein synthesis and promotes cellular health and growth (Manickam et al., 2016). Glucose-inhibited division protein (GidA), which is highly conserved in prokaryotes, serves as a tRNA modification enzyme and catalyzes the addition of a carboxymethylaminomethyl (cmnm) group at the 5′ position of the wobble uridine (U34) of tRNAs (Yu et al., 2019; Gao et al., 2020). GidA modification is evolutionarily conserved in bacteria and Eukarya, which is essential for efficient and accurate protein translation (Fislage et al., 2014). The disruption of *gidA* causes pleiotropy and affects multiple phenotypic traits. Therefore, the GidA-mediated tRNA modification pathway is thought to be the main regulatory mechanism of pathogenicity (Shippy and Fadl, 2014). The *gidA* gene is recognized to function in the regulation of bacterial growth, stress response, and virulence (Shippy and Fadl, 2014). In *Aeromonas hydrophila*, disruption of *gidA* resulted in altered cell morphology, reduced growth, and decreased cytotoxic enterotoxin production (Sha et al., 2004). In other bacteria genera, such as *Salmonella* spp. and *Streptococcus* spp., *gidA* mutants had motility defects, reduced survival under stressful conditions, and decreased expression of virulence proteins (Rehl et al., 2013; Zhang et al., 2014; Gao et al., 2016). In *Pseudomonas syringae*, the causal agent of bean spot disease, the *gidA* mutant had altered cell morphology and could not produce toxin (Kinscherf and Willis, 2002). In reality, GidA can regulate the expression of a variety of proteins at the translational level through tRNA modification, and thus can regulate the survival of bacteria in response to environmental signals under stressful conditions (Gustilo et al., 2008). Taken together, these studies highlight the importance of this conserved tRNA modification pathway in cellular processes. However, little is known about GidA in *L. capsici*.

*Lysobacter capsici* X2-3 was isolated from the wheat rhizosphere and showed marked antimicrobial activity against plant pathogenic fungi, oomycetes, and Gram-positive bacteria. Genes in the X2-3 genome were annotated using a combined analysis of the KEGG, COG, and GO databases, and several genes were predicted to be associated with antibiotic production (Yi et al., 2015). Although GidA family proteins play important roles in the regulation of bacterial growth, pathogenicity, and human diseases in pathogenic species, there are few studies on plant growth-promoting bacteria (PGPB). In this study, the biological function of *LC_GidA* was characterized by constructing an *LC_GidA* mutant. We demonstrated that the inactivation of *LC_GidA* significantly reduced bacterial growth, twitching motility, biofilm formation, root colonization, and stress response in *L. capsici* X2-3.

## Materials and Methods

### Bacterial Strains, Growth Conditions, and Plasmids

The bacterial strains and plasmids used in this study are listed in Table 1. Unless otherwise stated, *L. capsici* X2-3 and its derivative strains were grown at 28°C in nutrient broth (NB) medium or on NA (NB with 1.5% agar) medium. Transformants from the first crossover for the *LC_GidA* knockout were cultured on NBN (NB without 1% sucrose) or NAN (NBN with 1.5% agar) medium. Transformants bearing the second crossover were plated on NAS (NAN plus 10% sucrose) medium (Zou et al., 2011). All bacterial strains were incubated at 28°C. *Escherichia coli* strains were cultured in Luria-Bertani (LB) or LB plus 1.5% agar plates at 37°C. When necessary, the media were supplemented with the antibiotic ampicillin (Amp, 50 μg/ml), kanamycin (Km, 50 μg/ml), or gentamicin (Gm, 50 μg/ml), depending on the strains used.

**Table 1:** Bacterial strains, plasmids, and primers applied in this study.

Strains or plasmids	Relevant characteristics^*^	Source/Reference
*Lysobacter capsici*		
X2-3	Wide type strain	This study
MT16	The *gidA* deletion mutant of strain X2-3; *K*_m_^r^	This study
Com-16	The complemented strain of *gidA* deletion mutant	This study
*Escherichia coli*		
DH5α	*F*′ *recA*, Ф80 d lac*Z*, and ΔM15	TransGen
S17-1	Host strain for molecular cloning	This lab
*E. coli* Trans1-T1	*F*^−^φ80 (lacz) ΔM15ΔlacX74hadR (*r*_k_^−^, *m*_k_^−^) ΔrecA1398endA1tonA	TransGen
*Plasmids*		
pMD19-T Simple	Cloning vector; Amp^r^	Vazyme
pEASY-Blunt Simple	Cloning vector; *K*_m_^r^; Amp^r^	TransGen
PBBR1-MCS5	Broad-host-range vector with a *P_lac_* Promoter, *G*_m_^r^	Vazyme
pKMS1	6,400 bp, pUC18 polylinker, mob, oriV, and sacB; *K*_m_^r^	Zou et al., 2011
pKMS1-AB	pKMS1 carrying 0.998-kb gene fragment harboring two *LC_GidA* flanking regions (including the upstream and the downstream of *LC_GidA*); *K*_m_^r^	This study
pBBR1-*gidA*	pBBR1-MCS5 carrying 1.890-kb gene fragment harboring the intact *LC_GidA* gene; *G*_m_^r^	This study
PBBR1-*gfp*	pBBR1-MCS5 carrying 0.72-kb gene fragment harboring the intact *gfp* gene; *G*_m_^r^	This study
Primer	Sequence (5′-3′; restriction enzyme sites underlined)	Description
*gidA*up-F	5′-CGGGATCCCCTGAATGCTCCGCAAACTCT-3′	689 bp fragment flanking the left of *LC_GidA*
*gidA*up-R	5′-TCGGATCATATTCAGCGCTCGACGT-3′	
*gidA*down-F	5′-ACGTCGAGCGCTGAATATGATCCGA-3′	309 bp fragment flanking the right of *LC_GidA*
*gidA*down-R	5′-CCAAGCTTGAAGAACAGGCCCAGGTGGA-3′	
*gidA*F	5′-CGGAATTCGCTGAATGAACGATCCCTTCTAT-3′	1,890 bp *LC_GidA* gene
*gidA*R	5’-CGGGATCCTCACGCCACCCGCGAACGC-3′	
*gfp*F	5′-CGGAATTCATGGTGAGCAAGGGCGAG-3’	720 bp *gfp* gene
*gfp*R	5′-CGGGATCCTTACTTGTACAGCTCGTCCATGC-3′	

* *K*_m_^r^, kanamycin resistance; Amp^r^, ampicillin resistance; and Gm^r^, gentamicin resistance.

### Construction of the *LC_GidA* Deletion Mutant and Its Complemented Strain

The *LC_GidA* mutant was generated from the wild-type X2-3 strain by allelic homologous recombination. Briefly, two *LC_GidA* flanking regions were amplified by PCR using the primer pairs up F/R and down F/R (Table 1). The upstream and downstream PCR products were digested with *Bam*HI and *Hin*dIII, respectively. The digested fragments were ligated into the suicide vector pKMS1 (Table 1) to obtain the recombinant plasmid pKMS1-AB (Zou et al., 2011). The plasmid was transformed into X2-3 by electroporation. The *LC_GidA* mutant MT16 was obtained after two recombination events and confirmed by PCR and sequencing of the PCR products.

The fragment harboring the intact *LC_GidA* gene, which was amplified by PCR using the primers *gidA*F and *gidA*R (Table 1), was cloned into the expression vector pBBR1-MCS5 (Table 1) at the *Eco*RI and *Bam*HI site, resulting in the recombinant plasmid pBBR1-*gidA*, and then pBBR1-*gidA* was transformed into the mutant MT16 by electroporation (1.8 KV, 200 Ω, and 25 μF). The complemented mutant strain Com-16 was selected on NA plates with gentamycin (Kovach et al., 1994).

### Growth Curve Determination

The X2-3, MT16, and Com-16 strains were grown for 24 h at 28°C in NA medium and then inoculated into NB medium to OD_600_ = 1.0. The cultures were diluted 1:100 into NB medium. The strains were incubated at 28°C for 48 h with shaking at 180 rpm, and bacterial growth was examined every 4 h (Rehl et al., 2013).

### Motility Assay

The motility assay was performed as previously described (Rashid and Kornberg, 2000; Tomada et al., 2016). To test twitching motility, bacteria were grown for 24 h in NA medium at 28°C, and 3 μl of the bacterial cultures at a normalized OD_600_ were added to NYGB medium (0.6% agar) plates. The diameters of the areas occupied by the bacterial cells were measured after 3 days.

### Biofilm Formation Assay

The crystal violet technique was used to analyze the attachment of the different strains to an abiotic surface. The X2-3, MT16, and Com-16 strains were cultured in NB medium and adjusted to OD_600_ = 1. The cultures were diluted 1:100 into a glass tube containing 10 ml of NB medium supplemented with 1% sucrose or glucose. Then, the glass tubes were incubated at 28°C for 3 days with shaking at 180 rpm. The growth medium was removed, and the tubes were washed three times with sterile distilled water. Then, the glass tubes were stained with a 0.2% crystal violet solution for 10 min. The unbound crystal violet was removed, and the tubes were washed three times with sterile distilled water. Crystal violet was extracted with absolute ethanol, and the absorbance was measured at 575 nm (Zhang et al., 2018).

### Pellicle Formation

All *Lysobacter* strains obtained throughout the study were tested for their ability to produce biofilms, which were visualized as floating pellicle at the air–broth interface that completely blocked the surface of the culture and could not be dispersed by shaking. The X2-3, MT16, and Com-16 strains were grown in glass test tubes containing NB medium (with 1% sucrose or 1% glucose) at 28°C for 5 days without shaking (Latasa et al., 2012).

### Root Colonization Assay

Seven-day-old plants were collected, and the roots were cut into 1.5 cm segments. Fragments of uniform shape and size were placed into 96-well microtiter plate. Two hundred microliters of bacterial culture with an OD_600_ = 1.0 was added to the wells, and the plates were incubated at 28°C for 3 days. After the incubation period, the roots were removed from the cultures, washed with sterile water, and then added to 1 ml sterile water. The bacteria on the root surface were removed and dispersed in sterile water by shaking. One hundred microliters of the dispersed preparation was plated on NA agar and counted after 5 days (Tariq et al., 2014).

The plasmid pBBR1-*gfp* was transformed into the X2-3, MT16, and Com-16 strains by electroporation, and the transformants were selected on NA plates with gentamycin. The treatment was the same as above. To view the colonization of *L. capsici* X2-3-*gfp*, MT16-*gfp*, and Com-16-*gfp* on the root surfaces, the roots were observed using a confocal laser scanning microscope system (Zeiss LSM 800, Carl Zeiss AG, Jena, Germany) with an excitation wavelength of 488 nm. Images of at least 12 roots were obtained for each treatment (Liu et al., 2020).

### Stress Tolerance Assays

The bacterial strains were diluted 1:100 into NB medium, and experiments were conducted to test the OD_600_ under five environmental stresses. Stress treatments were applied as follows: for UV radiation, the cells were exposed to shortwave UV radiation (254 nm in a biological safety cabinet) at a distance of 60 cm for 45 min. For salt stress, NaCl was added to the bacterial cultures at final concentrations of 0.15, 0.25, and 0.35 mol/L (Li et al., 2014). For temperature stress, the cultures were incubated at 37 and 42°C with shaking at 180 rpm. Resistance against H_2_O_2_ was determined as described previously with slight modifications (Liu et al., 2019). H_2_O_2_ at concentrations of 0.1, 0.01, and 0.001 mM was added to the bacterial cultures and, the samples were incubated at 28°C for 10 min with shaking. After serially diluting the bacteria five times (10^−1^–10^−5^), 3 μl of each cell sample was dropped onto NA plates and incubated at 28°C for 3 days. The pH stress test was similar to the H_2_O_2_ test. The bacterium was serially diluted five times (10^−1^–10^−5^), and then 3 μl of each cell sample was dropped onto NA plates with pH values ranging from 5.0 to 9.0.

### RT–qPCR

The wild-type strain X2-3 and the mutant strain MT16 were cultivated until they reached an OD_600_ = 1. Total RNA was extracted using AG RNAex Pro Reagent [Accurate Biotechnology (Hunan) Co., Ltd.], and cDNA was synthesized by reverse transcription. Nineteen genes related to DNA replication, cell division, motility, and biofilm formation were chosen for RT–qPCR (Table 2). RT–qPCR experiments were carried out as instructed by the manufacturer [Accurate Biotechnology (Hunan) Co., Ltd.]. The 16S rRNA gene was used as an internal control (Qian et al., 2013). The relative transcription levels were calculated using the 2^–ΔΔCT^ method (Livak and Schmittgen, 2001).

**Table 2:** Primers used in RT–qPCR.

Primer	Sequence (5′-3′)	Description
16s rRNA-F	5′-GCTCGTGTCGTGAGATGTT-3′	RT–qPCR
16s rRNA-R	5′-TGTAGCCCAGGTCATAAGG-3′	
RT-*pilA*-F	5′-CAGCAAGGCTTTACCCTCATC-3′	RT–qPCR
RT-*pilA*-R	5′-TTCTTGGTGCGGATCGTGTAG-3′	
RT-*flgD*-F	5′-CGACCAGGAAGATTTCATCAAGC-3′	RT–qPCR
RT-*flgD*-R	5′-GATTGCTCCAGCGAGGTGAAC-3′	
RT-*fliF*-F	5′-CTCAACAACGAGGAGTTCAAGG-3′	RT–qPCR
RT-*fliF*-R	5′-TGGCTGGAATTGATCCGCTTG-3′	
RT-*flhB*-F	5′-GTTCACCACCCATCCGCTCAA-3′	RT–qPCR
RT-*flhB*-R	5′-CATCAGGCACTTGACCACCAG-3′	
RT-*fliQ*-F	5′-TTGGTCGTCGGTCTGCTGATT-3′	RT–qPCR
RT-*fliQ*-R	5′-AGCTTGGGCACGAAGGTCAAA-3′	
RT-*fliP*-F	5′-TGCCGCTGAAGATATTGCTGTT-3′	RT–qPCR
RT-*fliP*-R	5′-CGTCCAGTAACTGCTCAACAAGG-3′	
RT-*recN*-F	5′-AACTGATCCAGACCCATGCC-3′	RT–qPCR
RT-*recN*-R	5′-AATGCATCCTTGCCGATCCA-3′	
RT-*radC*-F	5′-CTGTTCCACGGCACCATCAA-3′	RT–qPCR
RT-*radC*-R	5′-AAATGATCGAGCAGGCGGAT-3′	
RT-*gyrA*-F	5′-CACCGGCAGCGATCTTATCT-3′	RT–qPCR
RT-*gyrA*-R	5′-GACAGCCAGTCGGAATGGAA-3′	
RT-*dnaA*-F	5′-GTGATGTACCTGCGTTCGGA-3′	RT–qPCR
RT-*dnaA*-R	5′-TGGCGCTTGAACTGATCCAT-3′	
RT-*rmuC*-F	5′-TGTCGAACGAGAAGTACCGC-3′	RT–qPCR
RT-*rmuC*-R	5′-TTCGACTTCTTCCTGCGCTT-3′	
RT-*n6amt-*F	5′-CCGGCGACATGGACTATCTG-3′	RT–qPCR
RT-*n6amt*-R	5′-GCACCAGGCTGGAATTGATG-3′	
RT-*ftsB*-F	5′-CTGGCTCGAGGATGACGGG-3′	RT–qPCR
RT-*ftsB*-R	5’-GTCAGGACGACGGTCGCATA-3′	
RT-*ftsQ*-F	5’-GTCGTCCGTTCCTGTACCTG-3’	RT–qPCR
RT-*ftsQ*-R	5′-AGTGACTGCCGTAACTGAGC-3’	
RT-*ftsI*-F	5′-ACCGTATTGCGCTTCGACAA-3′	RT–qPCR
RT-*ftsI*-R	5′-TCGAAGCTGACTTCGCTCAA-3′	
RT-*pgaA*-F	5′-GACGAACTGGTGATGCTCAAC-3′	RT–qPCR
RT-*pgaA*-R	5′-ATCGGCAGTTGGATGTTCTCG-3′	
RT-*pgaB*-F	5′-CGGTGCTCGGTTACGAATTGC-3′	RT–qPCR
RT-*pgaB*-R	5′-GAACGGATTGAGGCGGAAGGT-3′	
RT-*pgaC*-F	5′-CACCGAGGACATCGACATCAG-3′	RT–qPCR
RT-*pgaC*-R	5′-TTCAGGGTTTCAGGCATCAAGA-3′	
RT-*surA*-F	5′-CGAGGACGTGGTCAAGGAAA-3′	RT–qPCR
RT-surA-R	5′-CAGGAAGCGGTTCCACTCTT-3′	

### Statistical Analysis

All data were reported as mean standard at least triplicate experiments. The data were analyzed using the statistical SPSS software (version 18.0) by one-way ANOVA, and the mean was compared by Duncan’s multiple range test (DMRT) at the 5% probability level.

## Results

### General Analysis of GidA in X2-3

Glucose-inhibited division protein as a tRNA modification enzyme is highly conserved in bacteria and plays an important role in bacterial growth, stress response, and virulence (Shippy and Fadl, 2014). We conducted a search of the *L. capsici* X2-3 genome annotation (GenBank accession No. LBMI00000000.1) and observed that a potential ORF of approximately 1,890 bp in size was predicted to encode GidA (Supplementary Figure S1), which was named LC_GidA in *L. capsici*. BLAST analyses showed that the *LC_GidA* gene shares 62.43% identity with the *E. coli gidA* gene (GenBank accession No. NC_011750.1). The putative LC_GidA protein showed 63.81% identity with the *E. coli* GidA protein (GenBank accession No. YP_002410220.1; Supplementary Figure S1), which is a tRNA modification enzyme responsible for the proper biosynthesis of 5-methylaminomethyl-2-thiouridine (mnm5s2U) at position 5 of the wobble uridine (U34) of tRNAs.

### Deletion of *LC_GidA* Attenuates the Growth and Motility of X2-3

**Figure 1:**
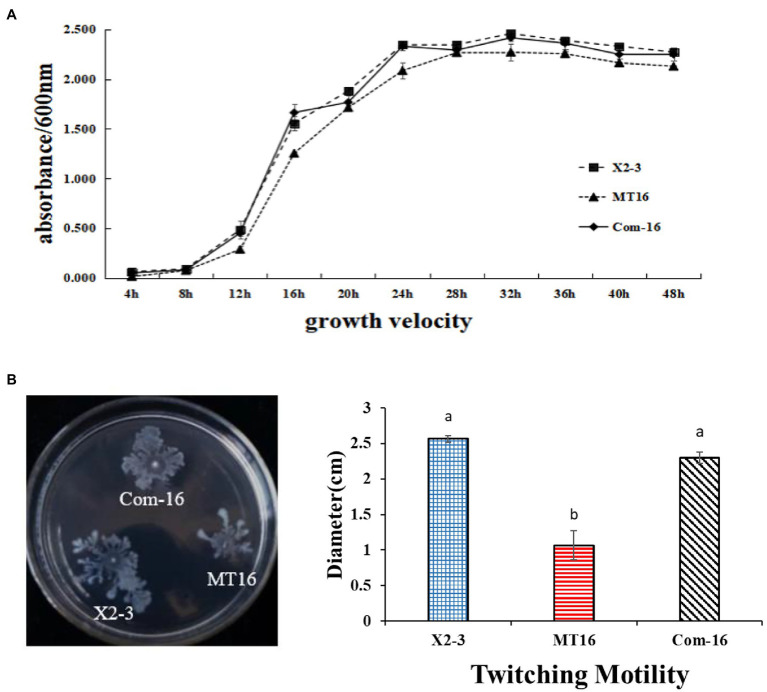
The growth and motility of X2-3, MT16, and Com-16. **(A)** X2-3, MT16, and Com-16 growth curves. The X2-3, MT16, and Com-16 strains were cultured in NB medium, adjusted to OD_600_ = 1.0, and then subcultured in fresh NB for 48 h. The OD_600_ values were tested every 4 h post-subculturing. All experiments were repeated at least three times. **(B)** Twitching motility of X2-3, MT16, and Com-16. The X2-3, MT16, and Com-16 strains were grown for 24 h in NB medium at 28°C and adjusted to OD_600_ = 1.0. Three microliters of each cell sample was dropped onto 0.6% agar plates for the motility tests. The diameters of each colony were measured after 3 days of incubation, and the resulting values were taken to indicate the bacterial motility. Each experiment was performed at least three times. a, not significant compared to X2-3. b, significant difference compared to X2-3.

To determine the function of the *LC_GidA* gene in *L. capsici* X2-3, a *LC_GidA* deletion mutant, termed MT16, was generated by integration of the pKMS1 plasmid (Supplementary Figure S2). The mutant was identified for the loss of 1,890 bp fragment coding region of the *gidA* gene by PCR with the primers *gidA*up-F and *gidA*down-R (Supplementary Figure S3). Additionally, the complemented mutant Com-16 was generated by insertion of the full-length *LC_GidA* into pBBR1-MCS5 and transfer of the resultant plasmid into MT16. The growth of wild-type strain X2-3 and the *LC_GidA* gene deletion mutant MT16 was assayed by measuring OD_600_ values from 4 to 48 h at 4 h intervals. As shown in Figure 1A, the cell density of MT16 was lower than that of X2-3 and Com-16, and the MT16 colony size was obviously smaller than that of X2-3 at the same timepoints. These results suggest that the loss of *LC_GidA* resulted in the attenuation of bacterial growth.

The twitching motility of X2-3 and the mutant MT16 were tested on 0.6% agar plates. After 3 days of incubation at 28°C, the diameter of the Com-16 complemented strain was 2.30 cm in NYGB media, very similar to the X2-3 wild-type strain (2.57 cm). In contrast, the MT16 *LC_GidA* mutant had decreased twitching motility significantly (Figure 1B). These results indicated that the *LC_GidA* gene is required for the motility of *L. capsici* X2-3.

### *LC_GidA* Is Involved in Biofilm and Pellicle Formation

**Figure 2:**
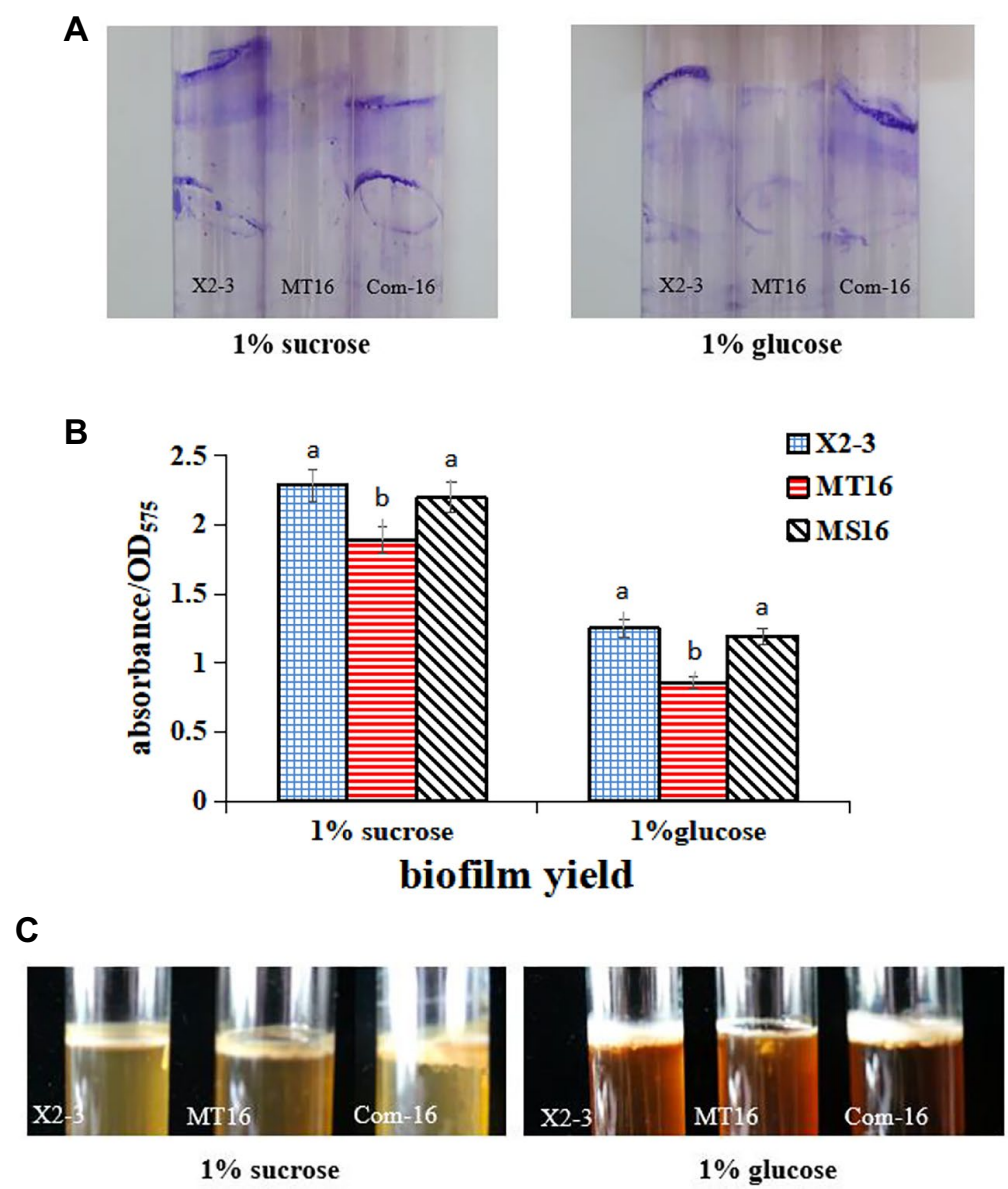
The ability to produce biofilms of X2-3, MT16, and Com-16. Biofilm formation of X2-3, MT16, and Com-16 on glass bottle surfaces after 3 days of incubation in NB medium supplemented with 1% sucrose or glucose. **(A)** Biofilm formation of X2-3, MT16, and Com-16 on glass bottle surfaces by crystal violet stain. **(B)** The results of the biofilm formation assays were quantified by measuring the absorbance of the crystal violet stain at 575 nm. Each experiment was performed at least three times. a, not significant compared to X2-3. b, significant difference compared to X2-3. **(C)** Pellicle formation by X2-3, MT16, and Com-16. All strains were analyzed after 5 days of incubation at 28°C, showing developed pellicles at the interface between the liquid and air in NB medium supplemented with 1% sucrose or glucose.

To measure the difference in the biofilm biomass of the MT16 and X2-3 strains, they were cultured in NB medium supplemented with 1% sucrose or 1% glucose for 3 days. The samples were then stained with crystal violet, and the biofilm biomass was quantified by measuring their OD_575_. Staining of bacterial cells with CV-staining showed that X2-3 and Com-16 produced much more biofilms of cell mass adhered to the glass surface than those produced by MT16 strain (Figure 2A). The biofilm biomass of MT16 was 17 and 30% lower than that of X2-3 in 1% sucrose and 1% glucose media, respectively. By contrast, the biofilm biomass of Com-16 was similar to that of the wild-type strain (Figure 2B). Furthermore, the pellicle, robust biofilm formed at the air–liquid interface of the culture, could be observed in 1% sucrose or 1% glucose NB medium after static culture for 5 days. The MT16 pellicle was much thinner than that of X2-3, both in 1% sucrose and 1% glucose NB medium, while pellicle formation was partially or fully restored in the Com-16 strain (Figure 2C). From these results we also determined that the rate at which X2-3 utilized different C sources varied, for example, the utilization rate of sucrose was higher than that of glucose; the utilization rate of glucose by the *LC_GidA* deletion strain was relatively low. These results indicated that deletion of the *LC*_*GidA* gene in MT16 decreased the biofilm biomass, while the Com-16 complemented strain recovered biofilm formation ability.

### Inactivation of *LC_GidA* Decreased the Colonization of *Lysobacter capsici* X2-3 on Wheat Roots

**Figure 3:**
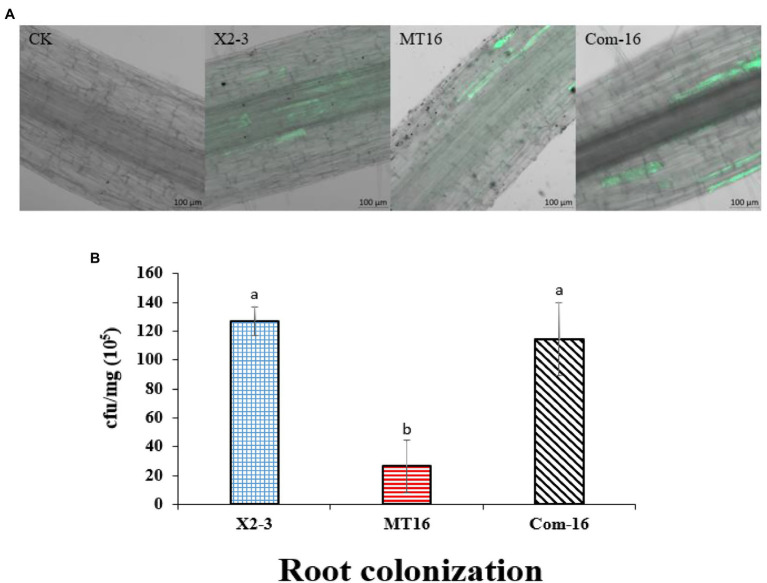
Qualitative and quantitative analysis of wheat root colonization by *Lysobacter capsici* X2-3 cells. The roots were cultured in X2-3, MT16, and Com-16 for 3 days. **(A)** Confocal scanning laser microscopy images of the roots colonized by *L. capsici*. Wheat roots without *gfp* inoculation as a control. Wheat roots colonized with X2-3*-gfp*, MT16*-gfp*, and Com-16*-gfp* for 3 days. Bar = 100 μm. **(B)** Quantitative analysis of root colonization by wild-type *L. capsici*, the *LC_GidA* deletion mutant and the complemented strain. a, not significant compared to X2-3. b, significant difference compared to X2-3.

Considering that the *LC*_*GidA* gene plays a role in biofilm formation, a quantitative measurement of root colonization was performed. Wheat roots were cultured in X2-3, MT16, or Com-16 for 3 days, and then 100 μl of the bacterial suspensions were plated on NA agar and cultured for 3 days. The results are shown in Figure 3B. The ability of the MT16 mutant to colonize wheat roots was significantly lower than that of the wild-type X2-3 strain; wheat root colonization was recovered in the Com-16 complemented strain. Green fluorescent protein-labeled X2-3, MT16, and Com-16 (X2-3-*gfp*, MT16-*gfp*, and Com-16-*gfp*) were used to detect the root colonization of *L. capsici* X2-3 under a confocal laser scanning microscope (Zeiss LSM 800, Carl Zeiss AG, Jena, Germany). GFP fluorescence shows successful colonization of X2-3 in root tip cells of wheat, the difference of colonization was determined by observing the GFP fluorescence area. As can be seen from Figure 3A, that the fluorescence area of the wild type is significantly larger than that of the mutant. The images showed that more X2-3-*gfp* cells were bound to the roots than MT16-*gfp* cells (Figure 3A). These results indicated that the inactivation of *LC_GidA* may affect the colonization of wheat roots.

### The *LC_GidA* Mutation Impairs Bacterial Resistance to Temperature, Salt, pH, and H_2_O_2_ but Has No Significant Effect on UV Radiation

**Figure 4:**
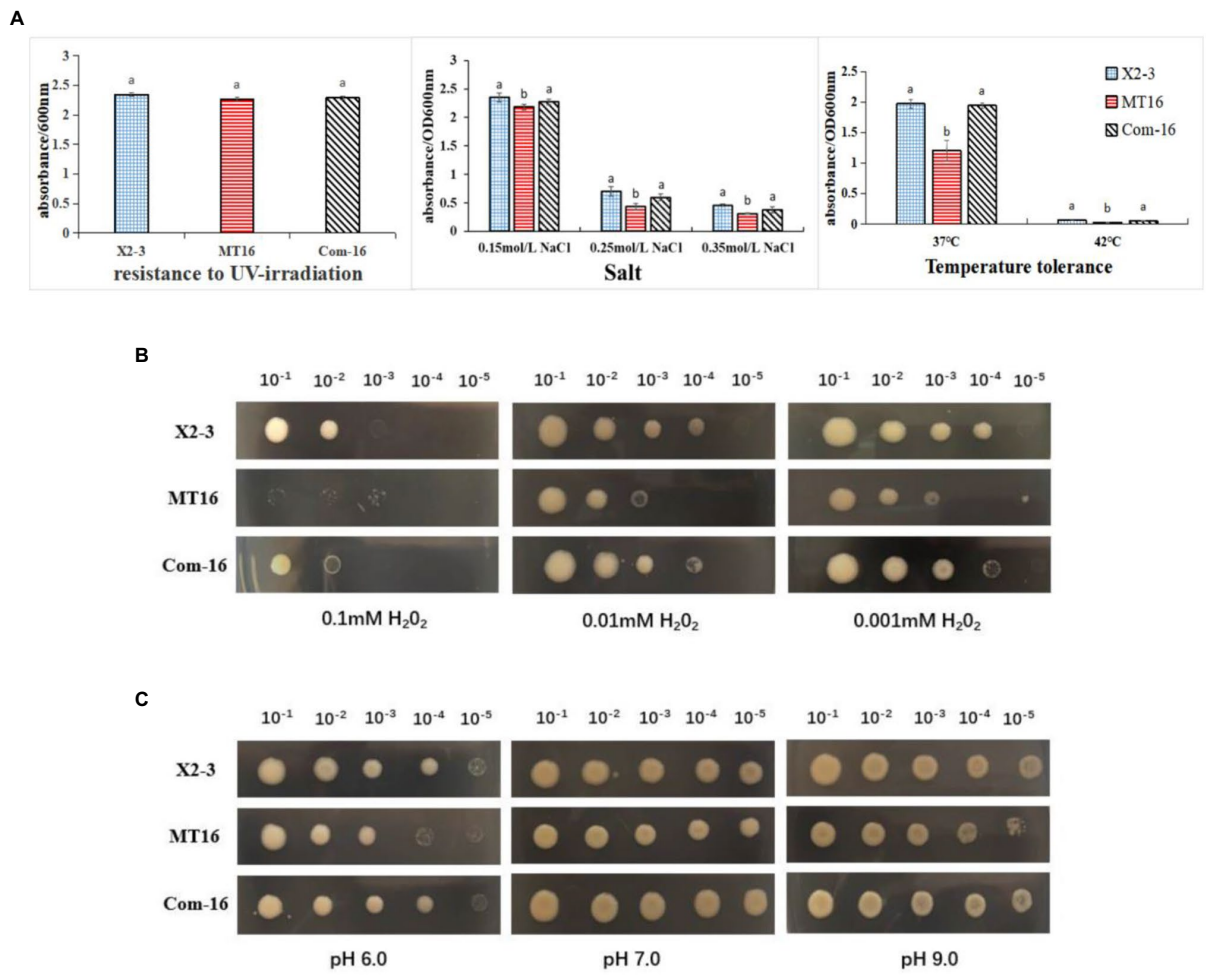
The survival under different stress conditions of X2-3, MT16, and Com-16. **(A)** Growth after 72 h of incubation of the wild-type X2-3, MT16 mutant, and Com-16 complemented strains after UV exposure for 45 min, at different concentrations of salt (0.15, 0.25, and 0.35 mol/L), and different temperatures (37 and 42°C). The results were quantified by measuring the absorbance at 600 nm. The data represent the means ± SDs of three independent experiments. a, not significant compared to X2-3. b, significant difference compared to X2-3. *LC_GidA* mutations impair resistance to **(B)** H_2_O_2_ and **(C)** pH in *Lysobacter capsici*. The wild-type X2-3, the mutant MT16, and the Com-16 complemented strains were grown on 0.1, 0.01, and 0.001 mM H_2_O_2_ **(B)** and at pH 6.0, pH 7.0, or pH 9.0 **(C)**. The bacterium was serially diluted five times (10^−1^–10^−5^). Three replicates for each treatment were used, and the experiment was repeated three times.

To assess the role of *LC_GidA* in stress tolerance, the growth yields of MT16, Com-16, and X2-3 were tested under different conditions, including temperature, salt, pH, and UV radiation. The growth of MT16 was significantly lower than that of X2-3 at 37 and 42°C, while Com-16 growth was basically restored to the level of the wild-type strain (Figure 4A). As shown in Figure 4A, the mutant had decreased survival at high osmotic pressure. When treated with UV radiation, there were no significant differences between the MT16 and X2-3 strains (Figure 4A). Compared with the wild-type strain, the growth of the mutant was inhibited at all concentrations of H_2_O_2,_ and the growth of Com-16 was also slightly affected under the high and low H_2_O_2_ conditions (Figure 4B). The pH resistance of *L. capsici* was significantly affected by the deletion of *LC_GidA* (Figure 4C).

### The *LC_GidA* Gene Regulates the Expression of Different Genes

**Figure 5:**
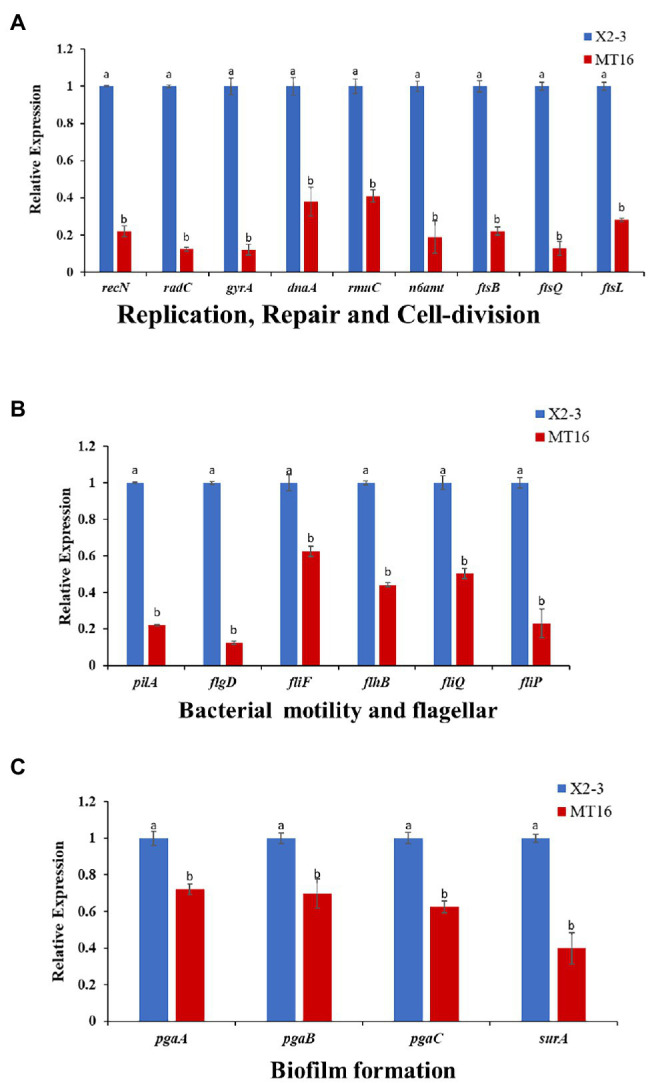
RT–qPCR of 19 selected differentially expressed genes. The X2-3 and MT16 mutant strains were cultivated to an OD_600_ = 1. RT–qPCR of 19 selected differentially expressed genes related to replication repair and cell division **(A)**, bacterial motility and flagellar formation **(B)**, and biofilm formation **(C)**. Three replicates for each treatment were used, and the experiment was repeated three times. Vertical bars represent SEs. a, not significant compared to X2-3. b, significant difference compared to X2-3.

To assess the role of *LC_GidA* as a global regulatory factor and further show that the deletion of *LC_GidA* leads to a decrease in growth, motility, and biofilm formation, 19 genes related to DNA replication, repair, cell division, motility, and biofilm formation in X2-3 were chosen for RT–qPCR. The results showed that the expression of genes related to motility, replication, cell division, and biofilm formation was significantly downregulated. The genes *radC*, *gyrA*, *recN*, *n6amt*, *dnaA*, *rmuC*, *ftsQ*, *ftsI*, and *ftsB*, which are related to DNA replication, repair, and cell division, were markedly downregulated in the *LC_GidA* mutant (Figure 5A). Six genes related to motility, *pilA*, *flgD*, *fliF*, *flhB*, *fliQ*, and *fliP*, were significantly decreased in the mutant compared with wild-type X2-3 (Figure 5B). Among the biofilm formation genes, four genes, *pgaA*, *pgaB*, *pgaC*, and *surA*, were significantly repressed in the *LC_GidA* mutant (Figure 5C).

## Discussion

Glucose-inhibited division protein, as an evolutionarily conserved tRNA modifying enzyme, catalyzes the addition of a cmnm group at the wobble uridine of tRNAs and is essential for proper and efficient protein translation (Fislage et al., 2014). GidA has exhibited important roles in regulating multiple biological processes, such as growth, cell division, and virulence in pathogenic bacteria (Shippy et al., 2011). However, the function in different bacterial species is not always the same. *L. capsici* is an effective biocontrol agents of plant diseases, and the role of GidA in *L. capsici* is unclear. In this study, we demonstrated that *gidA* affects cell growth, twitching motility, biofilm formation, root colonization, and stress response in *L. capsici* X2-3.

First, we obtained the *gidA* deletion mutant, we found that deletion of *LC_GidA* significantly reduced the growth and motility of *L. capsici* X2-3 (Figure 1), and this result is in agreement with previous reports on *E. coli* (Lies et al., 2015) and *Salmonella enterica* (Rehl et al., 2013). To further understand the regulatory effect of *LC_GidA*, nine genes related to growth, including six involved in DNA replication, recombination, and repair (*radC*, *gyrA*, *recN*, *n6amt*, *dnaA*, and *rmuC*), and three involved in cell division (*ftsQ*, *ftsI*, and *ftsB*), were analyzed in the *LC_GidA* mutant by RT–qPCR, and all of these genes were downregulated (Figure 5A). *GyrA*, *n6amt*, and dnaA are all related to DNA replication. *GyrA* is an essential gene that introduces negative supercoils into plasmid and chromosomal DNA (Rovinskiy et al., 2019); the *n6amt* gene encodes the main enzyme catalyzing the methylation of the adenine base (Zhang et al., 2016); and *dnaA* is the initiator of chromosomal DNA replication and has various activities in *E. coli* (Mizushima, 2000). RecN is a structural maintenance protein and is involved in RecA-mediated recombinational repair in *Deinococcus radiodurans* and *E. coli* (Uranga et al., 2017; Keyamura and Hishida, 2019). *RmuC* and *radC* function in recombination and repair *via* different mechanisms (Okaichi et al., 1995; Kosinski et al., 2005). Cell division is also essential in bacterial growth, and division regulated by the proteins FtsQ, FtsB, and FtsI is a key component in facilitating bacterial cell replication (Kureisaite-Ciziene et al., 2018). Taken together, these genes involved in DNA replication, recombination, repair, and cell division were all related to cell growth, and the downregulation of these genes in the *LC_GidA* mutant can explain the mechanism by which *gidA* disruption inhibits *L. capsici* X2-3 growth. Additionally, six genes related to motility, *pilA*, *flgD*, *fliF*, *flhB*, *fliQ*, and *fliP*, were downregulated in the *LC_GidA* mutant (Figure 5B). These RT–qPCR data related to replication, repair, cell division, and motility in the *LC_GidA* mutant strongly supported the biological results of attenuated cell growth and motility.

Deletion of *gidA* significantly reduced *L. capsici* biofilm formation and colonization of wheat roots. Biofilms attached to biological surfaces are indispensable for bacterial colonization and sessile growth (Kumara et al., 2017), and *gidA* is considered to play important roles in biofilm formation. In *S. mutans*, loss of *gidA* decreased the capacity for glucose-dependent biofilm formation by over 50% (Li et al., 2014). In our study, the deletion of *LC_GidA* attenuated biofilm formation in the *LC_GidA* mutant (Figure 2). This attenuation may be due to impaired growth of mutant MT16 or downregulation of genes associated with biofilm formation, or a dual function of impaired growth and downregulation of genes. Four genes, *pgaA*, *pgaB*, *pgaC*, and *surA* that were reported to be related to biofilm formation were tested by RT–qPCR. The results revealed that the genes *pgaA*, *pgaB*, *pgaC*, and *surA* were clearly downregulated in the *LC_GidA* mutant (Figure 5C). SurA is a major factor in the biogenesis of *β*-barrel outer membrane proteins, and the disruption of *SurA* in *S. enterica* serovar Typhi affects motility and biofilm formation (Lu et al., 2019). *PgaA*, *pgaB*, and *pgaC* have a profound role in the synthesis and secretion of poly-β-linked N-acetylglucosamine (PNAG), which has been characterized as a component of the bacterial surface responsible for biofilm formation in *E. coli* (Chen et al., 2014). Deletion of *pgaC* or *pgaB* dramatically reduced biofilms in *Klebsiella pneumoniae* and *Aggregatibacter actinomycetemcomitans* (Chen et al., 2014; Hathroubi et al., 2015; Shanmugam et al., 2017). Our results showed decreased biofilm formation and downregulated biofilm-related genes in the *LC_GidA* mutant, consistent with these studies. And the attenuation of biofilm formation in mutant can be explained by the downregulation of these genes. Biofilm formation is a determinant of the root colonization process in PGPBs, such as *Bacillus* (Chen et al., 2013; Xu et al., 2018). In our study, the *LC_GidA* mutant displayed an 80% reduction in bacterial colonization compared with X2-3 (Figure 3), suggesting that the *LC_GidA* gene is important for X2-3 colonization of wheat roots. Similar phenomena were found in a previous study with *B. velezensis* FZB42 (Al-Ali et al., 2018). In summary, the deletion of *LC_GidA* decreased X2-3 biofilm formation and colonization of the wheat rhizosphere.

In addition, biofilm formation is considered a generic mechanism for the survival of bacteria in stressful environments (Ansari and Ahmad, 2019; Gao et al., 2019; Masmoudia et al., 2019). As shown in Figure 4, the disruption of *LC_GidA* strongly reduced the growth of the mutant in high salt media, high temperature, different concentrations of H_2_O_2_, and different pH conditions. This result is in agreement with previous reports in *S. mutans* in which the *gidA* mutant showed a reduced ability to withstand stress conditions (Li et al., 2014). Moreover, in *Xanthomonas oryzae*, the *PXO_RS20535* mutant produced significantly less biofilm and had a clear diminution of growth and survival under stress conditions (Antar et al., 2020). These results indicated that biofilm formation may be involved in the growth of X2-3 in various stressful environments. Previous study proved that as a global regulatory factor, deletion of *gidA* significantly reduced the growth in most bacteria (Shippy and Fadl, 2014). In our study, growth curves showed that the *LC_GidA* mutant resulted in an attenuation of the bacterial growth rate compared with the wild type and entered the stationary phase at a slightly lower density. While the *LC_GidA* mutant grew more slowly, this relatively small difference is not sufficient to explain the dramatic biofilm formation and stress respond observed. In addition, despite the modest growth defect, the *LC_GidA* mutant did not show any deficiency in UV stress compared with the wild type. And RT–qPCR assays also eliminate the effect due to the growth deficiency of *LC_GidA* in regulating biofilm formation and stress response. Taken together, our study indicated that the *LC_GidA* mutant decreased biofilm formation and stress respond of X2-3.

In conclusion, this study demonstrated that *LC_GidA* regulates the expression of a series of genes involved in cell growth, twitching motility, biofilm formation, rhizosphere colonization, and stress resistance in *L. capsici* X2-3. The antimicrobial activity of the *LC_GidA* mutant against Gram-positive bacteria was also markedly decreased (Supplementary Figure S7). However, no significant changes in the antimicrobial activity of the *LC_GidA* mutant against either fungi or oomycetes were observed (Supplementary Figure S6), although deletion of *gidA* in pathogenic bacteria resulted in reduced pathogenicity. The regulatory mechanisms of *GidA* in antibacterial activity remain to be investigated. These findings provide new insights to better understanding the regulatory function of *gidA* in PGPB. This is the first report on the regulation of *LC_GidA* in *L. capsici*, as well as in the genus *Lysobacter*.

## Data Availability Statement

The datasets presented in this study can be found in online repositories. The names of the repository/repositories and accession number(s) can be found in the article/Supplementary Material.

## Author Contributions

DZ and HW conceived this study. DZ performed the mainly experiments, and some experiments were performed with the assistance of ZL and SH. DZ analyzed the data. DZ, CH, and AL wrote the manuscript. All authors contributed to the article and approved the submitted version.

## Funding

This work was supported by National Key R&D Program of China (grant number 2017YFD0201100) and Outstanding Youth Foundation of Shandong Province (grant number ZR2021YQ20).

## Conflict of Interest

The authors declare that the research was conducted in the absence of any commercial or financial relationships that could be construed as a potential conflict of interest.

## Publisher’s Note

All claims expressed in this article are solely those of the authors and do not necessarily represent those of their affiliated organizations, or those of the publisher, the editors and the reviewers. Any product that may be evaluated in this article, or claim that may be made by its manufacturer, is not guaranteed or endorsed by the publisher.
