# Supplementary material for: LC_Glucose-Inhibited Division Protein Is Required for Motility, Biofilm Formation, and Stress Response in Lysobacter capsici X2-3
Source: Front Microbiol. 2022 Mar 17;13:840792. doi: 10.3389/fmicb.2022.840792 (PMC8969512; doi:10.3389/fmicb.2022.840792)
Supplement: Supplementary file 1 [file Data_Sheet_1.docx]

Supplementary Material

## Supplementary Figures


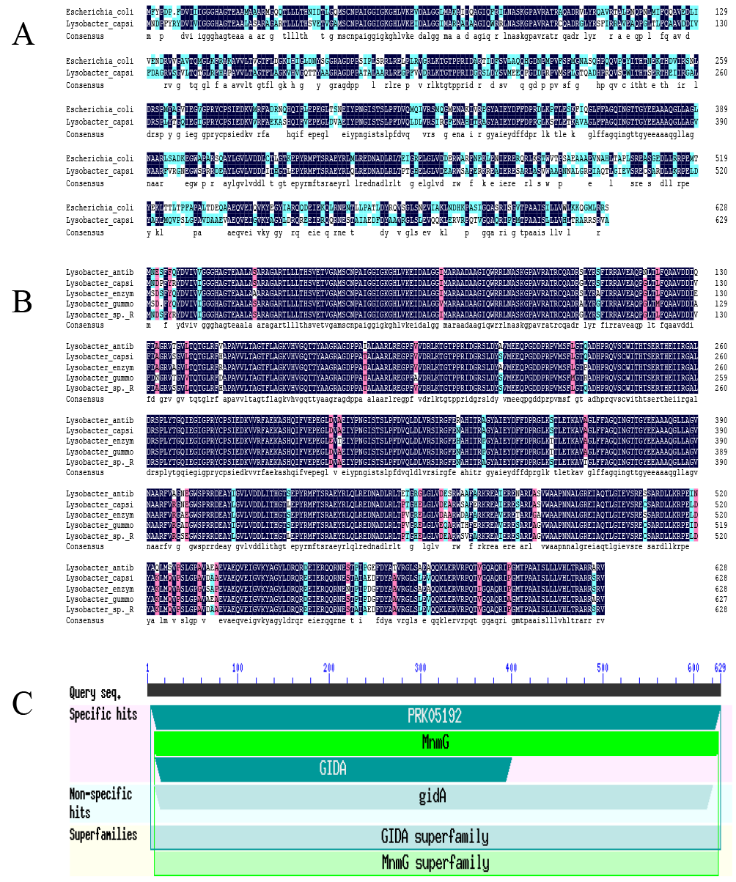


**Supplementary Figure 1.** Sequence analysis of the GidA in X2-3. **A:** Results of amino acid sequence in the full length of the GidA comparison between *Lysobacter capsici* X2-3 and *Escherichia coli* (GenBank accession No. YP_002410220.1) by DNAMAN. **B:** Multiple alignment of amino acid sequence in the full length of the GidA comparison between *L. capsici* X2-3 and *L. enzymogenes* (GenBank accession No. [WP_078996563.1](https://www.ncbi.nlm.nih.gov/protein/WP_078996563.1?report=genbank&log$=protalign&blast_rank=1&RID=ZZ0GBMZ9013)), *L. gummosus* (GenBank accession No. WP_057941129.1), *L*. sp. Root690 (GenBank accession No.WP_056109402.1), and *L. antibioticus* (GenBank accession No. [WP_057919558.1](https://www.ncbi.nlm.nih.gov/protein/WP_057919558.1?report=genbank&log$=protalign&blast_rank=1&RID=ZZ06YXE301R)) by DNAMAN. **C:** Prediction of *LC_GidA* by The Conserved Domain Database (<https://www.ncbi.nlm.nih.gov/cdd/?term>).


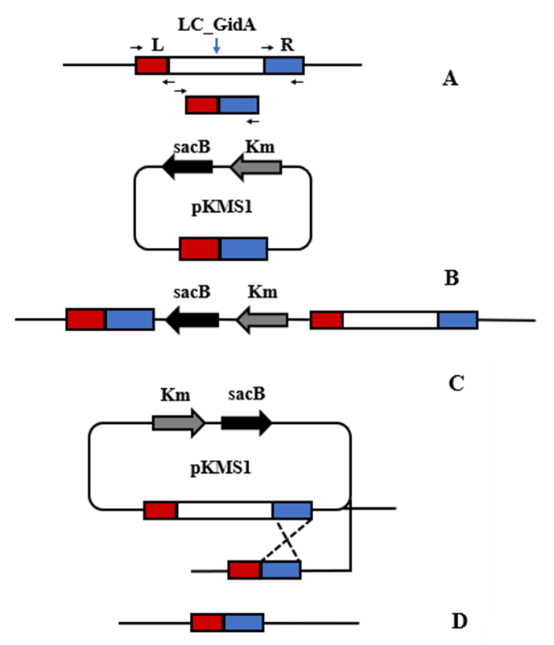


**Supplementary Figure 2.** Knockout mutagenesis of mediated by the suicide vector pKMS1 in *L. capsici* X2-3. **A:** The left and right flanks of *LC_GidA* were fused together by overlap PCR, and then ligated into the vector pKMS1. **B:** The first recombination exchange occurred on the left or right arm; **C:** The second recombination exchange on the 10% sucrose medium; **D:** the target gene was deletion. The shaded part indicates the left and right arm of the *LC_GidA* gene; the black arrow indicates the *SacB* gene; the gray arrow indicates the kanamycin gene (Zou et al., 2011).


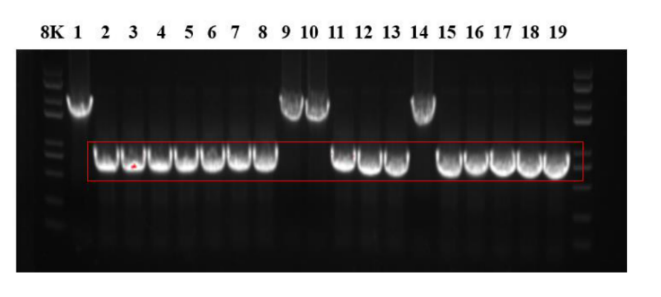


**Supplementary Figure 3.** Identification of mutant MT16. The PCR products were amplified using the primer *gidA*up-F and *gidA*down-R. The PCR products of wide type strain was 2888bp (1, 9,10,14), however the PCR products of mutant was 998bp (2-8, 11-13, 15-19). 8K Maker.


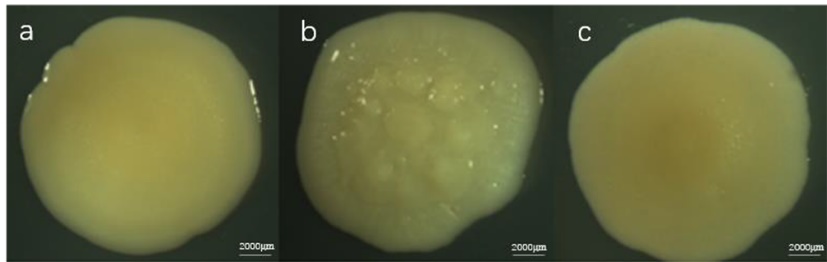


**Supplementary Figure 4.** Colony morphology of the wild-type X2-3 strain, the MT16 mutant and the Com-16 complemented strain after 3 days in NA medium. The experiment was performed at least in triplicate and representative results are shown. Scale bar, 2000 μm. a, The wild-type X2-3 strain; b, The MT16 mutant; c, The Com-16 complemented strain.


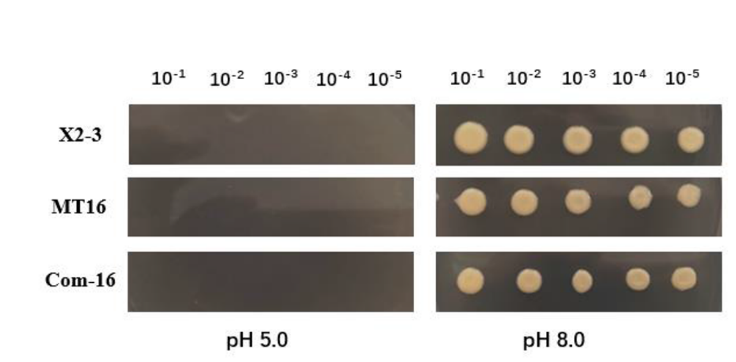


**Supplementary Figure 5.** The *GidA* mutation impaired resistance to pH in *L. capsici.* Three microlitres of the wild-type X2-3 strain, the MT16, and the Com-16 complemented strain were grown at pH 5.0 and pH 8.0 for 3 days. The bacterium was serially diluted 5 times (10^-1^, 10^-2^, 10^-3^, 10^-4^, 10^-5^). Three replicates for each treatment were used, and the experiment was repeated three times.

**
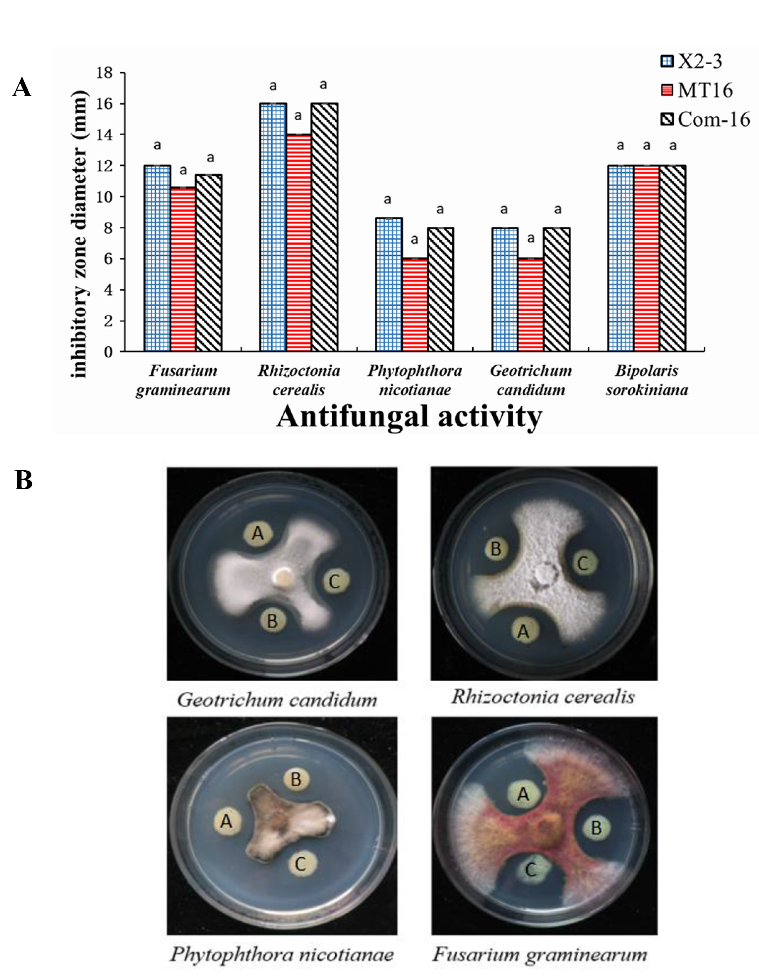
**

**Supplementary Figure 6.** *Fusarium graminearum*, *Rhizoctonia cerealis*, *Phytophthora nicotianae*, *Geotrichum candidum* and *Bipolaris sorokiniana* were used as indicators to test the antifungal activity. Three microlitres of each cell sample was dropped onto PDA for 2 days at 28°C.Then the fungi were inoculated in the center of the plate. Each experiment was performed at least three times. A, Wild-type strain X2-3; B, *LC_GidA* mutant MT16; C, Com-16 complemented strain. a, not significant compared to X2-3.

**
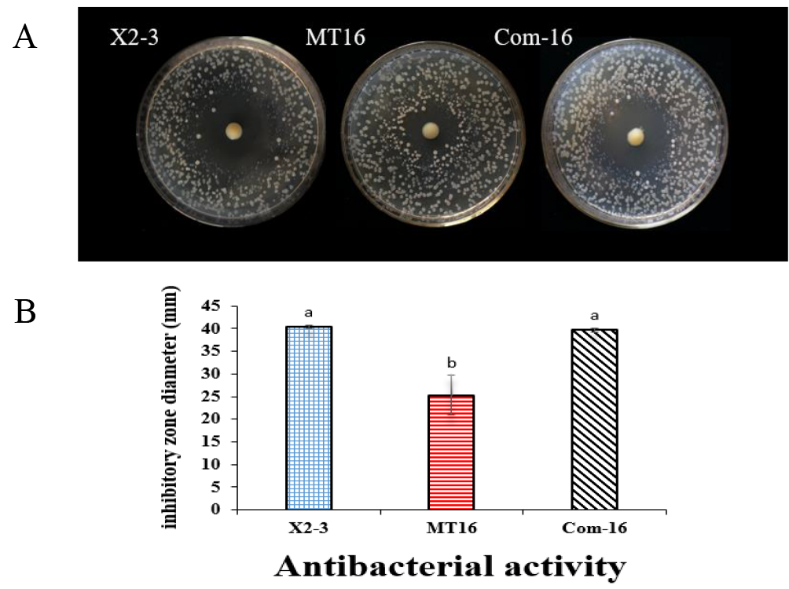
**

**Supplementary Figure 7.** *Paenibacillus polymyxa* was used as indicator to test the antibacterial activity. The X2-3, MT16 and Com-16 strains were cultured in NA medium for 2 days and then indicator bacteria were sprayed on the plate. The inhibition zones of each colony were measured after 24 h. Three replicates for each treatment were used, and the experiment was repeated three times. a, not significant compared to X2-3. b, significant difference compared to X2-3.


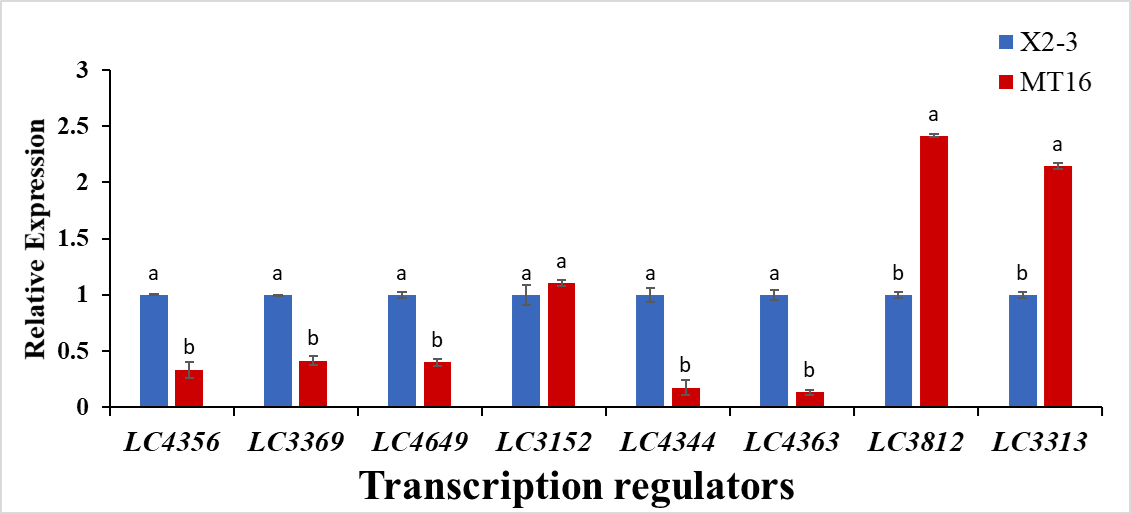


**Supplementary Figure 8.** RT–qPCR of 8 selected differentially expressed genes. The X2-3 and MT16 mutant strains were cultivated to an OD_600_=1, then RT–qPCR of 8 selected differentially expressed genes nearby *LC_GidA*. Three replicates for each treatment were used, and the experiment was repeated three times. Vertical bars represent standard errors. a, not significant compared to X2-3. b, significant difference compared to X2-3.
